# Research on the Reliability of Threshold Voltage Based on GaN High-Electron-Mobility Transistors

**DOI:** 10.3390/mi15030321

**Published:** 2024-02-25

**Authors:** Pengfei Dai, Shaowei Wang, Hongliang Lu

**Affiliations:** 1 Nanjing Electronic Devices Institute, Nanjing 210016, China; iamdaipengfei@163.com; 2 Key Laboratory for Wide Band Gap Semiconductor Materials and Devices of Education Ministry, School of Microelectronics, Xidian University, Xi’an 710071, China; wsw97382810@163.com

**Keywords:** GaN HEMT, *V*_TH_ drift, high temperature and high voltage

## Abstract

With the development of high-voltage and high-frequency switching circuits, GaN high-electron-mobility transistor (HEMT) devices with high bandwidth, high electron mobility, and high breakdown voltage have become an important research topic in this field. It has been found that GaN HEMT devices have a drift in threshold voltage under the conditions of temperature and gate stress changes. Under high-temperature conditions, the difference in gate contact also causes the threshold voltage to shift. The variation in the threshold voltage affects the stability of the device as well as the overall circuit performance. Therefore, in this paper, a review of previous work is presented. Temperature variation, gate stress variation, and gate contact variation are investigated to analyze the physical mechanisms that generate the threshold voltage (*V*_TH_) drift phenomenon in GaN HEMT devices. Finally, improvement methods suitable for GaN HEMT devices under high-temperature and high-voltage conditions are summarized.

## 1. Introduction

The high-electron-mobility transistor (HEMT) is a heterojunction field-effect transistor that generates a conducting channel through the formation of a high concentration of two-dimensional electron gas (2DEG) at the heterojunction, thus realizing the conduction of the device [1,2,3]. At the same time, GaN material has a strong, spontaneous, and piezoelectric polarization effect; this characteristic can improve the density and mobility of 2DEG in the HEMT structure [4]. So, GaN-based HEMTs have become a hot spot in the field of RF/Microwave Filtering and power switching devices.

A lot of breakthroughs have been made in research targeting GaN HEMT devices. Miller et al. [5] designed a temperature-dependent ASM-HEMT for modeling GaN HEMTs at elevated temperatures that can accurately capture DC and RF measurements collected at different temperatures. Sahebghalam et al. [6] studied and proposed a physically based analytical model for HEMTs that can be operated continuously from room temperature to high temperatures in both linear and saturated states. Khan et al. [7] theoretically and experimentally analyzed the temperature dependence of 2DEG for AlGaN/GaN HEMTs and AlGaN/InGaN/GaN pHEMTs. Wu et al. [8] studied the time-resolved threshold voltage (*V*_TH_) instability of 650-V Schottky-type gate GaN HEMTs under high-temperature gate bias conditions. By applying forward and reverse bias conditions, it was found that the *V*_TH_ of GaN HEMTs shifted negatively under high-temperature forward gate bias and positively under high-temperature reverse gate bias. Nuo et al. [9] proposed a time-resolved extraction method for studying the *V*_TH_ evolution of Schottky-type gate GaN HEMTs biased at a high *V*_DS_. The gate heterostructures of p-GaN gate HEMTs are Schottky-type gates and ohmic-type gates [10,11]. The Schottky-type contact formation causes the p-GaN body to electrically float [12], which leads to the threshold voltage (*V*_TH_) instability problem. The ohmic-type gate HEMT has also been found to exhibit less severe *V*_TH_ instability after stress [13], but it requires large gate drive currents and specially designed gate drive circuits. Wei et al. [12] studied the *V*_TH_ evolution of GaN HEMTs with a drain-induced dynamic *V*_TH_ shift in GaN HEMTs with Schottky gate contacts and explained the underlying mechanism with a charge storage model. Tang S. W. et al. [14] investigated gate current characteristics to explain the phenomenon of threshold voltage shift in AlGaN/GaN HEMTs with p-GaN gates. The threshold voltage shift was observed by applying a positive gate bias for a set stress time, and consistent trap energy levels with activation energies of EA ~ 0.6 eV were found. Tang S. W. et al. [15] reported the capacitance-dependent stability of *V*_TH_ in p-GaN power HEMTs under high dVG/dt conditions. It was also found that the charging capacitance due to displacement current directly affects the *V*_TH_ stability in high dVg/dt events. Zhou X. et al. [16] investigated the effect of total ionizing doses (TIDs) on the dynamic *V*_TH_ in p-GaN HEMTs. A non-monotonic dependence of *V*_TH_ on dynamic gate stress was observed, indicating the mechanism of the effect of irradiation damage on metal/p-GaN/AlGaN. Finally, the variations in gate current, drain leakage, and gate capacitance were provided in order to verify the mechanism. Chen J. et al. [17] investigated the device stability of p-GaN gate HEMTs under self-heating effects using the on-drain current injection technique. By analyzing the gate leakage current as well as simulating the TCAD of the electrically heated device, it was shown that the self-heating-induced *V*_TH_ instability is electron trapping in the p-GaN gate stack. In order to analyze the *V*_TH_ degradation mechanism of AlGaN/GaN HEMTs with hot-electron stress (HES) in the semi-conducting state, Lin J. H. et al. [18] proposed a complete *V*_TH_ mechanism model. By analyzing the characteristic curves of drain current versus gate voltage (*I*_D_-*V*_G_), it was found that *V*_TH_ shifts in the positive direction after stress. However, an instability of *V*_TH_ in the positive direction was observed even after recovery. In addition, light experiments and Silvaco simulations were performed to verify the accuracy of the proposed model. However, the problem of GaN HEMT *V*_TH_ drift still has not been effectively addressed. Threshold instability seriously affects the switching speed of GaN power devices, which introduces unpredictable timing delays and reduces the power switching efficiency to the point of failure. Therefore, the study of GaN HEMT threshold drift and its mechanism is necessary.

In this paper, a review of previous work is presented. The existing methods for analyzing *V*_TH_ drift are studied. The variation rule of *V*_TH_ under the switching stress conditions of GaN HEMT devices is analyzed, and the variation rule of *V*_TH_ under variable temperature conditions of GaN HEMT devices is also analyzed. Finally, a method for effectively improving the structure of GaN HEMT devices is presented.

## 2. Factors Affecting *V*_TH_ Drift

In RF power-switching circuit applications, with a low on-resistance and high transconductance of the device, GaN HEMT devices fully open only the conducting channel at high gate voltages [19]. To ensure that the gate voltage is in a safe operating range, the magnitude of the gate leakage current needs to be small. As seen in Table 1 [20], the safe operating gate voltage range of a GaN HEMT that satisfies the above two conditions is only 1 to 2 V. Therefore, the drift in *V*_TH_ not only affects the switching speeds and the characteristic parameters of GaN HEMTs but also influences the safe gate voltage range of the devices. Therefore, GaN HEMT threshold instability is a critical issue that needs to be solved to promote the application of GaN HEMTs in power circuits and systems.

### 2.1. Effect of Switching Stress

**Figure 1:**
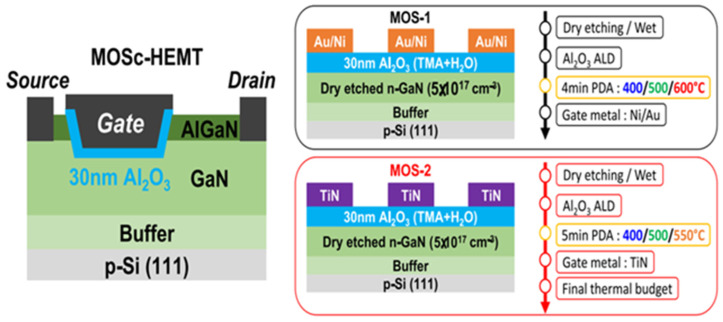
Schematic diagram of MOSc HEMT and a schematic diagram and process flow of MOS-1 and MOS-2 [24].

**Figure 2:**
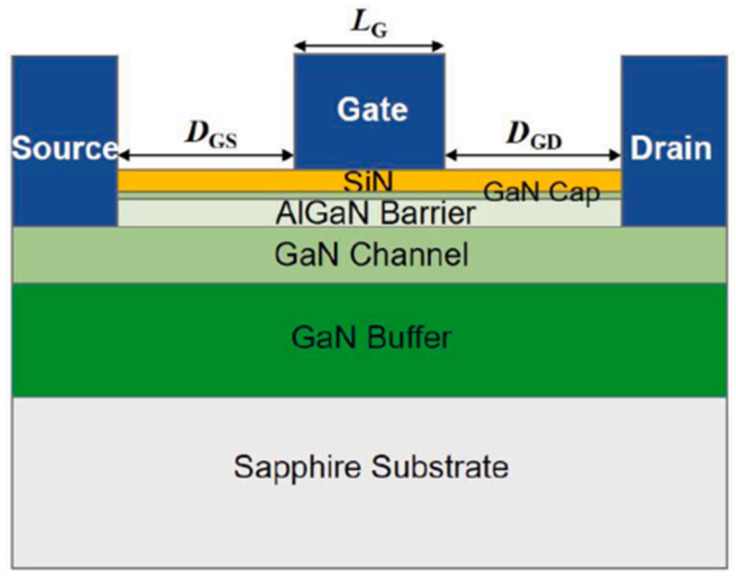
Cross-section of the proposed MIS-HEMT with a SiN passivation layer [25].

**Figure 3:**
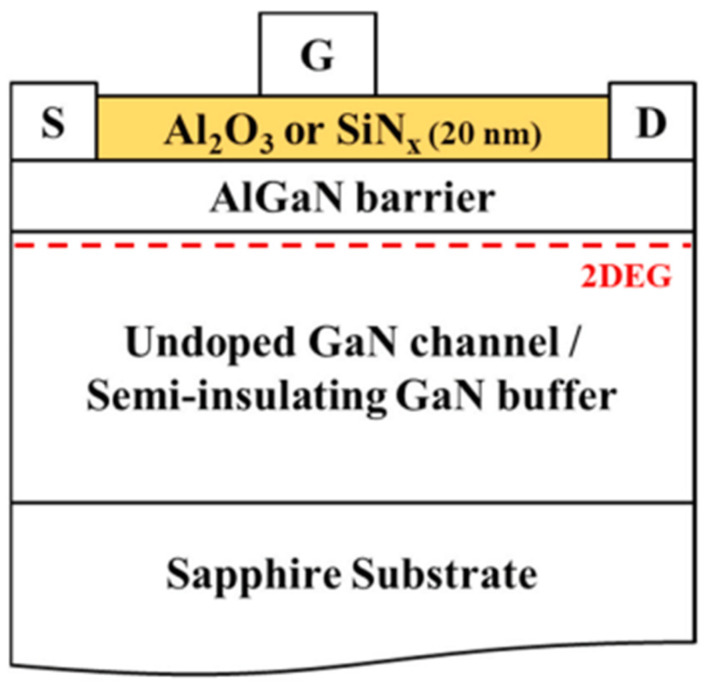
Different gate dielectric layers.

Nowadays, GaN HEMTs exist in various device structures, as shown in Figure 1, Figure 2 and Figure 3 [24,25,26]. When the device is under switching stress conditions, the body defects and interface defects in the device may capture electrons or holes. When the release of electrons or holes from deep energy level defects does not keep up with the switching response frequency, it causes the charge distribution in the gate region of the GaN HEMT power device to change, leading to a change in *V*_TH_ as well [27].

**Figure 4:**
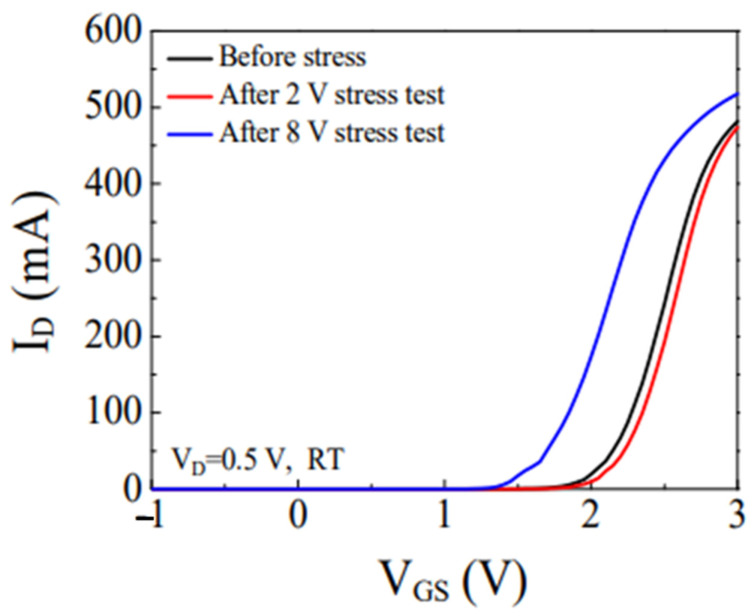
*I*_D_-*V*_G_ characteristics of a p-GaN gate HEMT before and after different gate voltage stress tests at room temperature [28].

Myeongsu Chae et al. [28] investigated the degradation of a p-GaN gate induced by forward gate voltage stress in a GaN HEMT with a Schottky-type p-GaN gate. The results of the study are presented in Figure 4. A positive shift in *V*_TH_ occurred during the stress test at *V*_G_ = 2 V. However, a negative offset of *V*_TH_ was observed during the stress test at *V*_G_ = 8 V.

Wang et al. [20] measured the pulse transfer characteristics of GaN HEMT devices with *V*_DS_ set at 1 V. Additionally, to assess the stability of *V*_TH_ under forward gate bias voltage, the pulse transfer characteristics were measured at positive *V*_GSQ_ (up to 8 V) and *V*_DSQ_ (0 V). The *V*_DSQ_ value of 0 V was intended to allow for an even distribution of the 2DEG channel voltage to ensure symmetry in the gate toward the source and drain sides. In this experiment, it was observed that the value of *V*_TH_ increased as the *V*_GSQ_ increased_._

**Figure 5:**
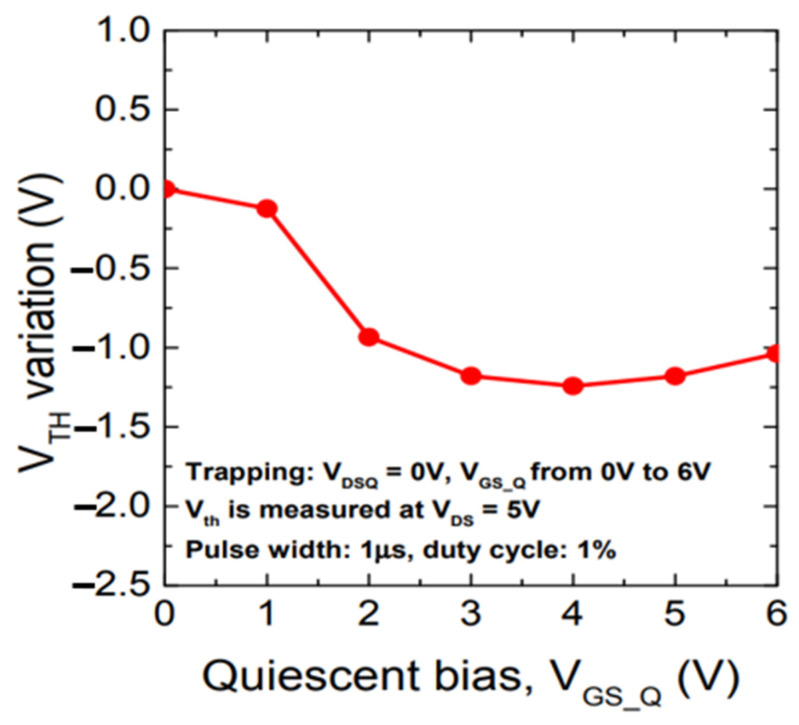
The trend in the threshold voltage change [29].

Meneghini et al. [29] measured the pulse transfer characteristics of a GaN HEMT device for a *V*_DS_ value of 5 V. As shown in Figure 5, it was found that the value of *V*_TH_ decreased with an increase in *V*_GSQ_.

Loizos Efthymiou et al. [30] investigated the threshold voltage instability that occurs in p-GaN-gated AlGaN/GaN-on-Si HEMT devices under off-state drain stress. The threshold voltage drift could increase by up to 40% under drain bias conditions of less than 50 V. This was attributed to the phenomenon where, under off-state conditions, high drain bias conditions caused the Mg receptor to ionize, generating a positive *V*_TH_ offset charge through contact with the gate. However, the increase in threshold voltage at drain biases greater than 50 V appeared to saturate.

Luca Sayadi et al. [31] investigated the effect of gate contact on threshold voltage stability in p-GaN gate GaN HEMTs using double-pulse measurements of p-GaN gate devices. It was found that in the case of high-leakage Schottky contacts, the accumulation of holes in the p-GaN region leads to a negative shift in the threshold voltage. On the contrary, in the case of low-leakage Schottky contacts, hole depletion in the p-GaN region caused a positive threshold voltage shift.

**Figure 6:**
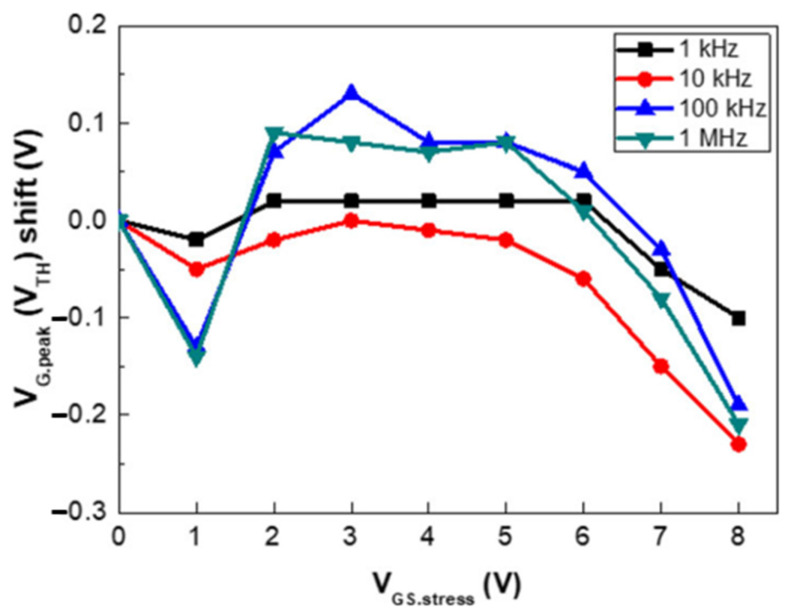
*V*_TH_ offset relative to different gate stress voltages [32].

Lai Y. C. et al. [32] investigated the shutdown state characteristics of 650 V-enhanced p-GaN gate AlGaN/GaN HEMTs after applying different levels of gate stress bias. As shown in Figure 6, the threshold voltage exhibited a positive bias at low gate stresses and a negative bias at gate stresses greater than 6 V.

**Figure 7:**
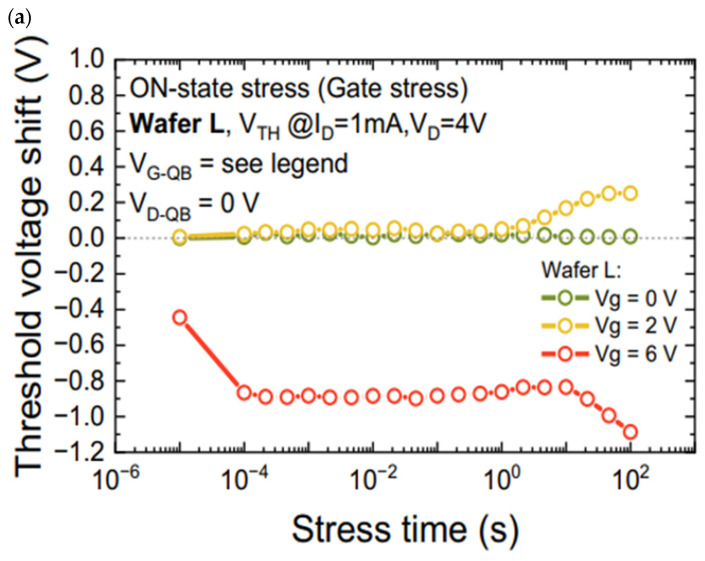

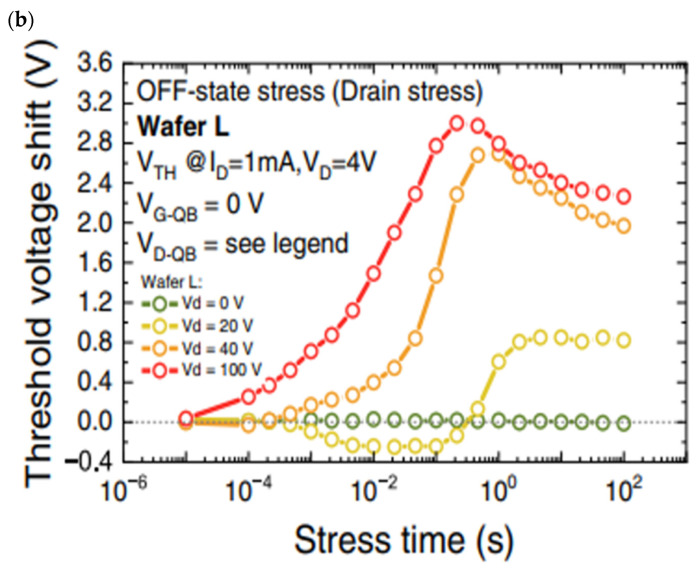
Threshold voltage drift case: (**a**) low gate voltage and (**b**) high gate voltage [33].

Favero D. et al. [33] investigated in detail the effect of electronic gate leakage on the stability of the threshold voltage of normally turned-off GaN HEMTs using p-GaN gates. The analysis was based on a combination of DC, pulsed, and transient measurements conducted on two test wafers with different gate processes, leading to varying levels of gate leakage currents. In Figure 7, the drift results of the threshold voltage for the different cases are shown.

From the above discussion, it is evident that the gate drive voltage can cause a GaN HEMT device to experience current collapse and *V*_TH_ instability. It is also observed that the off-state bias stress affects not only the dynamic on-resistance but also the stability of the threshold voltage. The shift in *V*_TH_ of the Schottky contact at the p-GaN gate under high- and low-leakage conditions is also investigated. Negative threshold voltage offsets can result in false conduction, which can significantly impact the overall performance of the circuit. Positive threshold voltage offsets can affect the on-resistance of the device, leading to a degradation in the performance of the power switching circuit. Next, the reasons for these phenomena will be analyzed and discussed.

### 2.2. Effects of Thermal Stress

**Figure 8:**
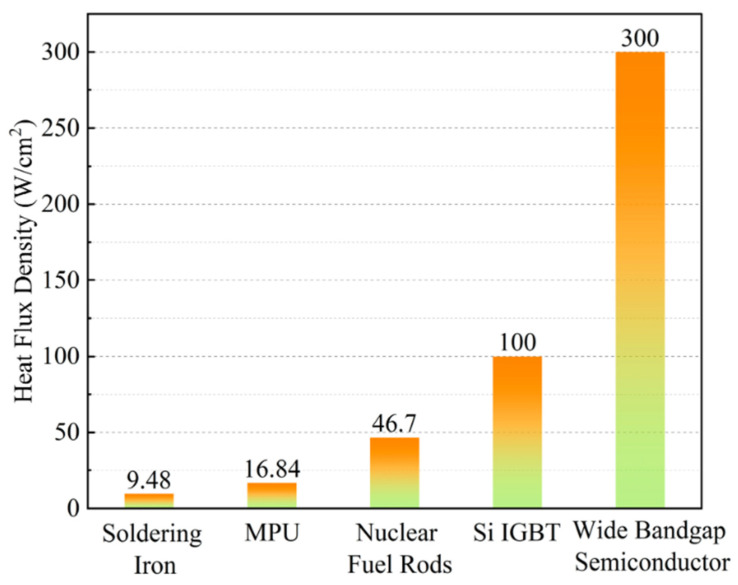
Heat flux density of different heat sources [36].

At present, p-GaN gate HEMT devices are widely used in high-power applications, such as fast chargers and universal power supplies (UPSs) [34,35]. As the chip area continues to shrink, devices become more and more compact, leading to an increase in their temperature. In Figure 8, the heat flux density of various heat sources is displayed. It can be seen that the heat flux density of the GaN HEMT is three times higher than that of silicon-based IGBTs [36]. Therefore, in order to enhance the gate stability and reliability of p-GaN gate HEMTs, it is essential to investigate the temperature dependence on *V*_TH_ instability and its underlying physical mechanisms.

**Figure 9:**
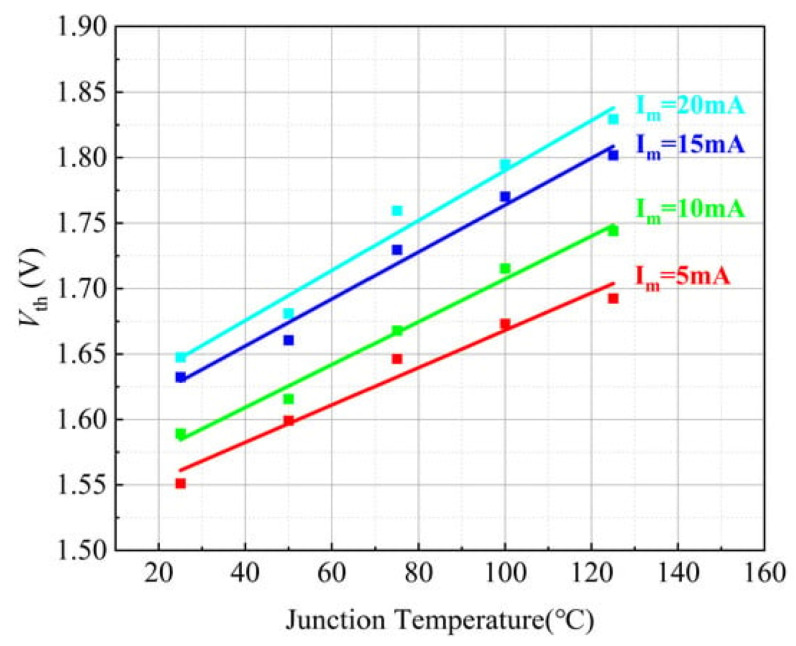
Relationship between *V*_TH_ and junction temperature under different current conditions [36].

Kaihong Wang et al. [36] investigated the variation rules of saturation voltage with low current injection, threshold voltage, and diode voltage drop with temperature for a GaN-HEMT. In Figure 9, the Vth-Tj curves are displayed for various currents. It can be seen from the figure that Vth increases with temperature at a constant current.

Hao Wu et al. [8] investigated time-resolved threshold voltage instability under high-temperature gate bias. Under the application of high-temperature forward gate bias conditions (HTGB), the threshold voltage of a p-GaN-AlGaN/GaN-HEMT showed a negative shift, which could be attributed to the defects present in the AlGaN layer. Under the application of high-temperature reverse gate bias conditions (HTRB), the threshold voltage of the device exhibited a positive shift. This shift was attributed to the accelerated movement of holes in the p-GaN layer and electrons trapped within the AlGaN layer at elevated temperatures, which in turn accelerated the inter-particle complexation. This also indicated that the positive threshold voltage shift under HTRB showed a positive correlation with temperature.

Under thermal stress conditions, Wang et al. [37] studied the phenomenon of *V*_TH_ shift in GaN HEMTs based on an ohmic-type gate and a Schottky-type gate. The variation in *V*_TH_ shift with temperature was tested for both Schottky-type gate devices and ohmic-type gate devices when *V*_DS_ was set to 5 V. The GaN HEMT device with a Schottky-type gate showed an increase in the value of *V*_TH_ as the temperature increased. The GaN HEMT device with an ohmic-type gate showed a decrease in the value of *V*_TH_ as the temperature increased.

**Figure 10:**
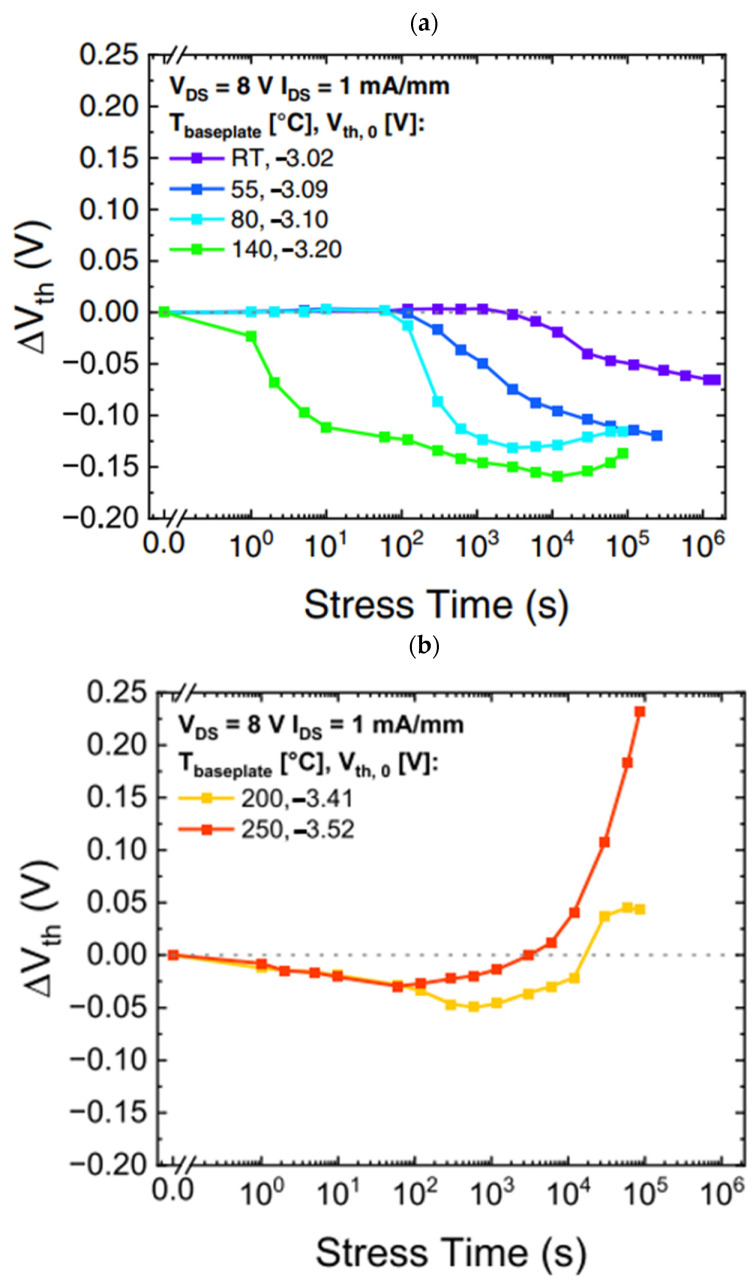
Threshold voltage shift [38]: (**a**) negative shift and (**b**) positive shift.

Pilati M. et al. [38] investigated the effects of low- and high-temperature operating life (LTOL and HTOL) of GaN HEMTs for RF applications. Based on several stress experiments conducted at various temperature levels, the presence of two degradation modes—negative and positive shifts in the threshold voltage—was demonstrated. The results of the threshold voltage shift are presented in Figure 10.

Based on the above analysis, the threshold voltage exhibited a negative drift phenomenon under the HTGB condition. Under the HTRB condition, the threshold voltage exhibits a positive drift phenomenon. Not only that, but the difference in threshold voltage drift between Schottky gate devices and ohmic gate devices occurs under varying temperature conditions. The devices vary in their performance based on changes in the operating environment. The effect of temperature difference on the threshold voltage of the devices is also an important factor to study. In the next section, the effect of temperature on the threshold voltage of devices and variations among different devices will be analyzed and summarized.

## 3. Reasons for *V*_TH_ Drift

*V*_TH_ drift is affected by switching and thermal stresses. However, there is uncertainty regarding the direction of the *V*_TH_ drift, which significantly limits the application of GaN HEMT devices. Therefore, there is a need to investigate the *V*_TH_ drift phenomenon in GaN HEMT devices.

### 3.1. Analysis of Switching Stresses

In order to analyze the gate voltage distribution of GaN HEMT devices, Tallarico et al. [39] proposed a schematic diagram illustrating the equivalent diode distribution of GaN HEMT gates. In this experiment, it was found that the GaN HEMT gate mainly consists of two back-to-back diodes in series. When *V*_G_ > 0 V, Schottky diode D1 is reverse-biased, and the p-GaN/AlGaN/GaN diode D2 is forward-biased. The voltages across diodes D1 and D2 are denoted as *V*_1_ and *V*_2_, respectively. The gate voltage (*V*_G)_ is the sum of *V*_1_ and *V*_2_.

In order to analyze the mode of electron and hole transport at the gate of GaN HEMT devices, the Space Charge-Limited Conduction (SCLC) model was utilized to simulate the gate current pattern in relation to the voltage.

When the GaN HEMT conducting channel is not open (OFF region, *V*_G_ < *V*_TH_), the gate voltage *V*_G_ is mainly supported by diode D2. The voltage value of *V*_2_ is much larger than that of *V*_1_.

When *V*_TH_ < *V*_G_ < *V*_SAT_ (ON-I area), the conducting channel of the GaN HEMT is not yet saturated. The negative charge near the gate of the device increases. As the negative charge repels the channel electrons, it results in a positive shift in the *V*_TH_ of the GaN HEMT.

When *V*_SAT_ < *V*_G_ < *V*_GT_ (ON-II area), the conducting channel of the device is saturated. When the voltage across diode D2 saturates, the main gate voltage is then carried by diode D1. The negative charge near the gate increases, causing the *V*_TH_ of the device to continue drifting forward.

When *V*_G_ > *V*_GT_, holes are injected from the gate metal, and some of them cross the potential barrier into the channel. This process leads to an accumulation of positive charge near the gate. When *V*_G_ is much larger than *V*_GT_, many holes are injected, causing the *V*_TH_ of the device to drift negatively.

In order to validate the methodology’s accuracy, Yuanyuan Shi et al. [40] examined the off-state gate current (*I*_G_-*V*_D_) and leakage current (*I*_D_-*V*_D_) of GaN HEMTs when *V*_G_ is set to 0 V. The experiment revealed that when *V*_GSQ_ is less than 6 V, the off-state leakage current *I*_D_-*V*_D_ curves of GaN HEMT devices after the initial scan align with the pre-stress off-state leakage current curves. When *V*_GSQ_ exceeds 6 V, the gate stress triggers the GaN HEMT device to switch on, leading to the injection of holes from the gate into the GaN channel. After the gate stress dissipates, the holes injected into the GaN channel combine with electrons, resulting in a significant increase in *I*_D_. In this experiment, it was observed that the off-state *I*_D_-*V*_D_ curve during the second scan almost perfectly aligns with the pre-stress *I*_D_-*V*_D_ curve. This indicates that the holes injected into the channel attract channel electrons, causing the *V*_TH_ to shift negatively.

In order to investigate the effect of gate contact on threshold voltage stability, Luca Sayadi et al. [31] fabricated ohmic-gate and Schottky-gate p-GaN HFETs. The results of double-pulse gate stress measurements and device simulations were analyzed and compared between the two types of gates. This analysis was conducted using double-pulse gate stress measurements and extensive device simulations. In the case of ohmic gate contacts, *V*_TH_ was consistently negative and increased in magnitude with higher gate stress voltage. In the case of Schottky gate contacts, the threshold voltage offset was nonlinear with the applied gate stress voltage. In their study, it was found that the variation in the threshold voltage was mainly related to the hole tunneling current through the Schottky barrier and the hot ion current through the AlGaN barrier. To verify the reliability of the experiments, a negative threshold voltage shift was tested and found in the case of a high-leakage Schottky contact. A positive threshold voltage shift was tested and found in the case of a low-leakage Schottky contact. The proposed theory effectively explains the experimental phenomenon.

**Figure 11:**
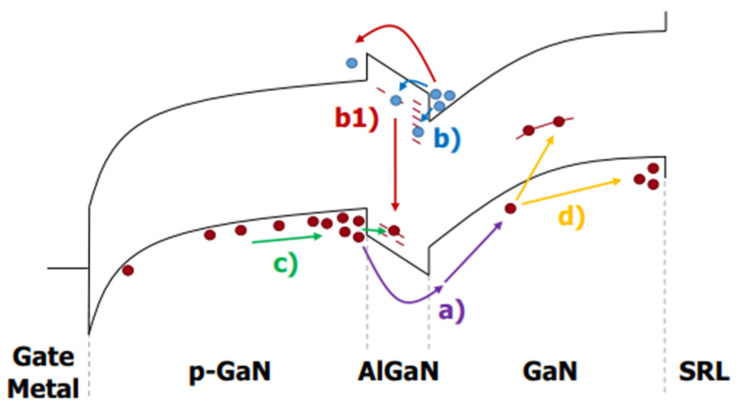
Mechanism of device threshold voltage instability [32].

In Figure 11, the mechanism of the device’s threshold voltage instability is shown. The symbol (a) indicates hole depletion in p-GaN, leading to a positive *V*_TH_ drift. The symbol (b) indicates electron trapping at the AlN/GaN interface and/or in the AlN trap, suggesting a positive *V*_TH_ drift. The symbol (c) denoted hole accumulation in 2DHG and the subsequent hole trapping in AlGaN, leading to negative *V*_TH_ drift. The symbol (d) indicates hole trapping in the GaN buffer, leading to a negative drift in *V*_TH_. The symbol (b1) represents the mechanism that occurs in the wafer to stabilize the *V*_TH_. This also indicates the presence of four different charge-trapping processes, whose interactions determine the sign and magnitude of the threshold voltage change. A moderate increase in gate leakage helped eliminate negative threshold voltage instability under positive gate bias and significantly reduced positive threshold shift under off-state stress.

Based on the above discussion, the phenomenon of threshold voltage drift in GaN HEMTs under forward gate stress is analyzed for the first time. The gate voltage is divided into three regions, and the gate current is divided into electron and hole currents. The movement in the threshold voltage at different stages is analyzed using the SCLC theory. It is summarized that when 0 V < *V*_G_ < 6 V, the main reason for the forward drift in the threshold voltage is the predominant electron capture in the AlGaN barrier. At 6 V < *V*_G_ > 9 V, the negative shift in the threshold voltage is primarily due to dominant hole capture in the AlGaN barrier and hole injection into the GaN buffer. In addition, the impact of high-leakage Schottky contacts and low-leakage Schottky contacts on the threshold voltage drift is also analyzed. Under gate stress, the device experiences negative threshold voltage drift in the case of high-leakage Schottky contacts due to the accumulation of holes in the p-GaN region. On the contrary, in the case of low-leakage Schottky contacts, the negative threshold voltage drift is hole depletion in the p-GaN region. Overall, the varying leakage of the device under gate stress conditions results in a modification of the total charge stored in the p-GaN region, leading to a shift in the threshold voltage.

### 3.2. Analysis of Thermal Stress

In order to analyze the relationship between the ohmic-type gate devices, the Schottky-type gate devices, and *V*_TH_ drift, Wang et al. [37] tested and analyzed the relevant characteristics of ohmic gate devices and Schottky gate devices.

In the experiments, the characteristic parameters of an ohmic-type gate device were demonstrated. The I-V characteristics between two contact pads with different channel distances were shown. The test revealed a knee point voltage, which may have been caused by the surface of the GaN material being affected during the fabrication of the device [37]. It was shown that the presence of the knee voltage (*V*_Knee_) makes the effective bias on the p-i-n junction (*V*p-i-n) smaller than the applied gate bias (*V*_Bias_), which, in turn, affects the conduction of the ohmic-type gate device. The voltage values of *V*_Knee_ under various temperature conditions were displayed. It could be visualized that in ohmic-type devices, the value of *V*_Knee_ decreases with increasing temperature. The relationship between *V*_PIN_ and temperature was demonstrated. It was found that the value of *V*_PIN_ varies less with different temperatures. *V*_TH_ comprised *V*_Knee_ and *V*_PIN_. Therefore, the ohmic-type gate device showed a negative shift in the *V*_TH_ under high-temperature conditions.

In the experiment, the characteristic parameters of the Schottky-type gate device were demonstrated. It was found that when the bias voltage is within the range of 0–2 V, most of the applied gate bias voltage is taken by the P-I-N junction. At bias voltages greater than 2 V, the MS junction begins to absorb some of the voltage. As the bias voltage exceeds 4.5 V, the MS junction begins to bear more of the bias voltage than the P-I-N junction. It was also observed that as the temperature increases, the *V*_MS_ shows an increasing trend, while the *V*p-i-n shows a decreasing trend. It was shown that *V*p-i-n decreases as the temperature increases at a constant gate bias. A trend of gradual increase in *V*_TH_ with increasing temperature was observed. This indicates that the device needs to apply a larger bias voltage to the gate to maintain sufficient *V*p-i-n and open the channel at high temperatures. This was the reason why the Schottky-type gate device showed positive *V*_TH_ drift at high temperatures.

For 650 V Schottky-type p-GaN gate HEMT devices, Hao Wu et al. [8] conducted forward and reverse bias experiments under various temperature conditions. Their study also investigates how changes in temperature affect the threshold voltage offset under reverse gate bias conditions. It was summarized and found that under HTFB conditions, the tunneling effect induced by trapped cavities leads to a negative *V*_TH_ drift, which is independent of the temperature. Under HTRB conditions, the positive *V*_TH_ drift is primarily influenced by temperature. This was mainly due to the trapping of electrons by defects created through hole emission and hot holes. Also, the higher the temperature, the greater the positive *V*_TH_ drift.

**Figure 12:**
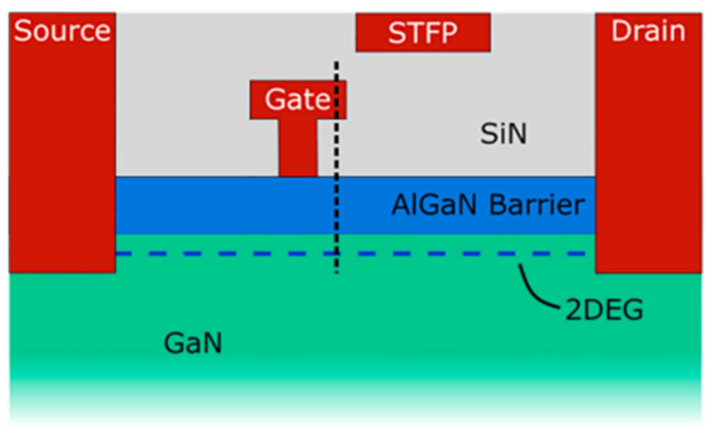
Simplified structure of the DUT [38].

**Figure 13:**
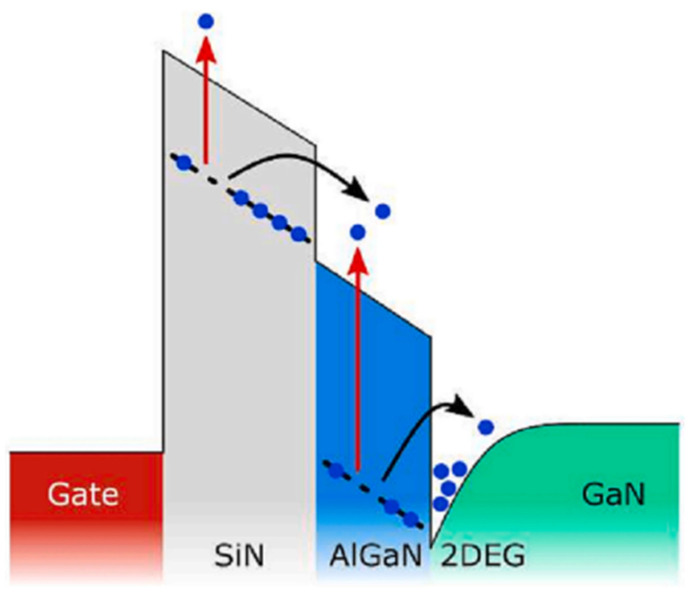
Band diagram at the gate stack, with temperature and field-assisted electron de-capture indicated by red and black arrows, respectively [38].

**Figure 14:**
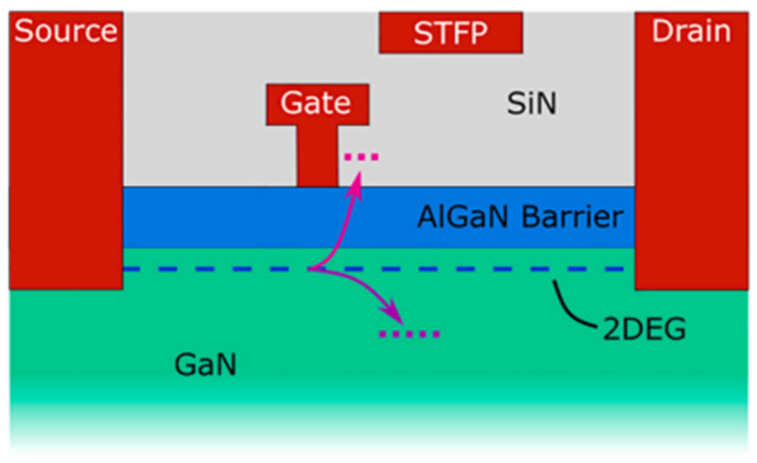
Visual representation of the hot electron capture location during the process [38].

Pilati M. et al. [38] found that a negative *V*_TH_ shift was attributed to the temperature and magnetic field-assisted electron de-trapping of the passivated/aluminum nitride stack under the gate, in agreement with previous reports. A positive *V*_TH_ shift occurred only at high temperatures and was due to the trapping of the passivated/aluminum nitride stack under the gate. In Figure 12, Figure 13 and Figure 14, the movement of hot electrons through the device structure is depicted.

In summary, Schottky-type gate devices exhibit a positive *V*_TH_ shift at high temperatures. This shift is primarily attributed to the rise in gate current caused by high temperatures. The increase in gate current results in a higher bias voltage across the Schottky metal/p-GaN junction in the device, consequently shifting the threshold voltage positively. A negative *V*_TH_ shift occurs in ohmic gate devices at high temperatures. This shift is primarily caused by the increase in hole injection at high temperatures, leading to a decrease in the inflection voltage of the ohmic metal/p-GaN contact in ohmic devices. Consequently, this results in a negative shift in *V*_TH_. Meanwhile, when applying forward and reverse bias voltages to Schottky gate devices under high-temperature conditions, it is observed that the threshold voltage of the Schottky gate p-GaN devices shifts negatively under forward gate bias. It is found that defects in the AlGaN layer trap the accumulated 2D hole gas in the p-GaN layer near the p-GaN/AlGaN interface. It can be determined that the negative shift in the threshold voltage under HTFB is independent of temperature. However, the threshold voltage of Schottky-gated p-GaN devices exhibits a positive shift under reverse gate bias. It is found that defects generated by thermal cavities at high temperatures trap hole emissions in the p-GaN layer and electrons within the AlGaN layer. Thus, the conclusion that the positive threshold voltage shift at HTRB is temperature-dependent is obtained.

## 4. Conclusions and Future Perspectives

**Figure 15:**
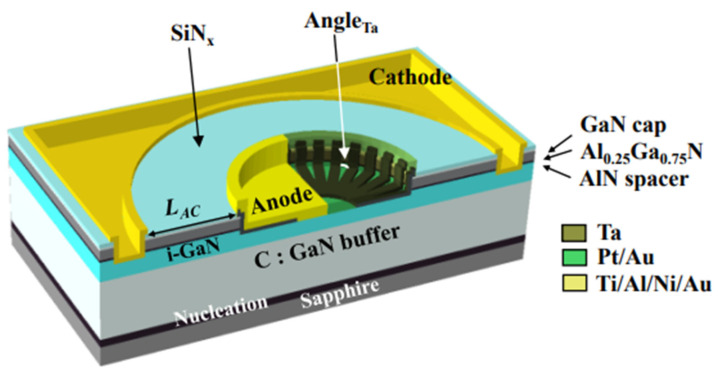
Schematic cross-section of an AlGaN/GaN lateral SBD with a DBA structure on sapphire [43].

**Figure 16:**
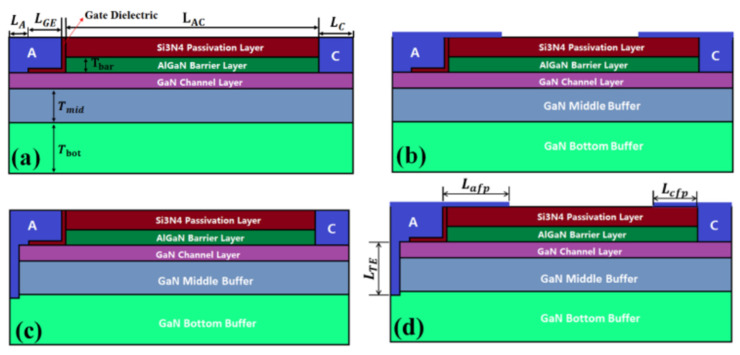
Cross-section: (**a**) SBD with gate edge termination, (**b**) SBD with gate edge termination in combination with a field plate, (**c**) T-type anode located deep in the buffer layer at the bottom of the SBD, and (**d**) T-anode in combination with a field plate located deep in the bottom buffer layer of the Schottky barrier diode [42].

**Figure 17:**
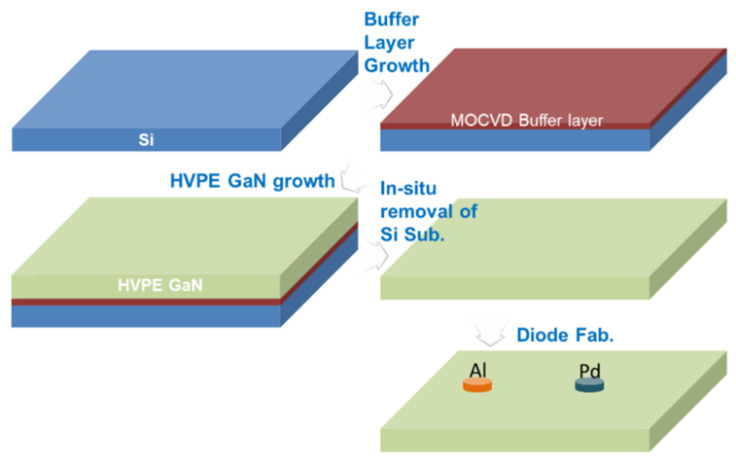
The preparation process of a Pd/Si-based FS-GaN Schottky diode [43].

**Figure 18:**
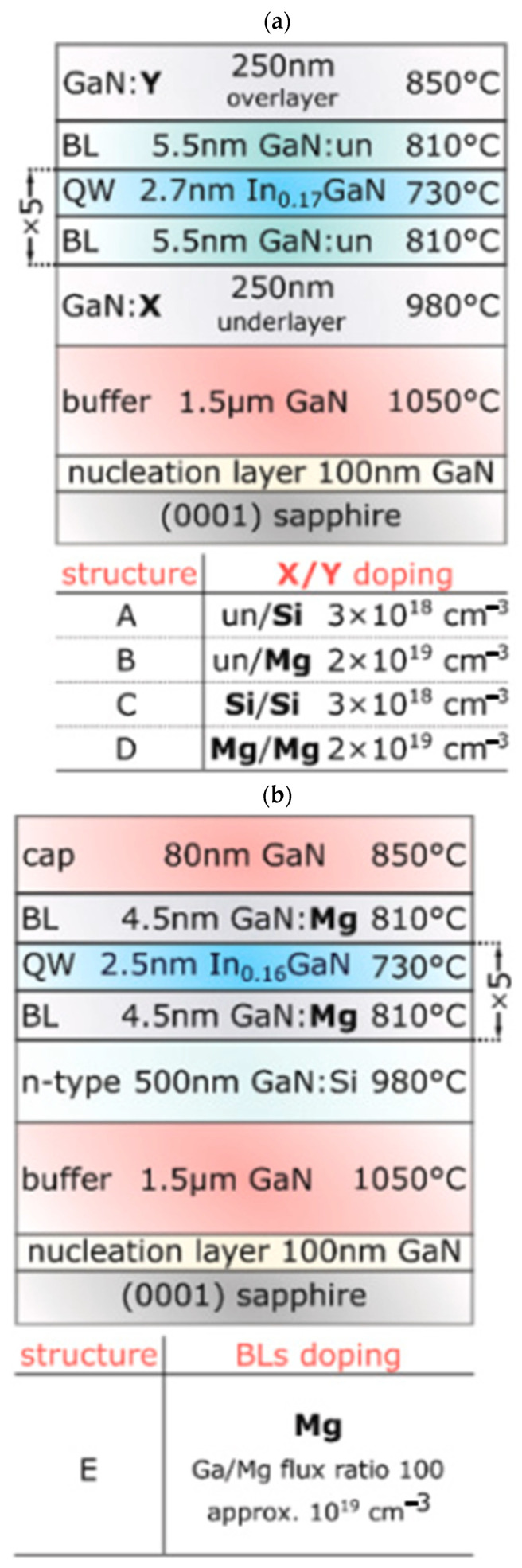
Schematic representation of an epitaxial structure with (**a**) different doping in the lower and upper GaN layers and (**b**) a Mg-doped barrier layer [44].

In this paper, a review of previous research is presented. The relationship between switching stress and *V*_TH_ drift is analyzed and discussed. It is also stated that the reverse turn-on of the gate Schottky junction plays a significant role in the rapid increase in the gate current in GaN HEMT devices. To minimize on-resistance, it is necessary to operate a GaN HEMT device in the ON-II area. Therefore, the optimization of the GaN HEMT gate structure aims to increase the gate voltage range between channel saturation and gate Schottky junction turn-on. The instability in the threshold voltage instability of p-GaN gate devices is examined using double-pulse measurements on Schottky-contacted p-GaN devices and compared with device simulations. It is shown that the threshold voltage depends on the balance between the hole-tunneling current through the Schottky barrier and the thermionic current through the AlGaN barrier. This balance may result in a change in the total charge stored in the p-GaN, ultimately leading to a shift in the threshold voltage. The effect of the AlGaN back-barrier is also investigated, and the results show an almost permanent but limited negative threshold voltage shift due to hole accumulation at the channel/back-barrier interface. The following optimization methods can be used to optimize the device structure: 1. The selection of a suitable metal is crucial to increase the height of the resulting metal/p-GaN Schottky barrier [41,42,43]. Figure 15 shows a Schottky barrier diode (SBD) structure with a double-barrier design that was proposed to achieve a low conduction AlGaN/GaN SBD and an ultra-high breakdown voltage. In Figure 16, different SBD-type devices with the same physical dimensions are shown. In Figure 17, a detailed manufacturing procedure is shown. 2. Optimization of the p-GaN layer thickness and doping concentration to increase the voltage withstand capability of the layer [44]. In Figure 18a, details of the doping in a GaN layer are shown. In Figure 18b, an 80 nm thick undoped GaN layer is shown. 3. Insertion of a thin layer of AlN between the p-GaN/AlGaN layers to restrict hole injection.

**Figure 19:**
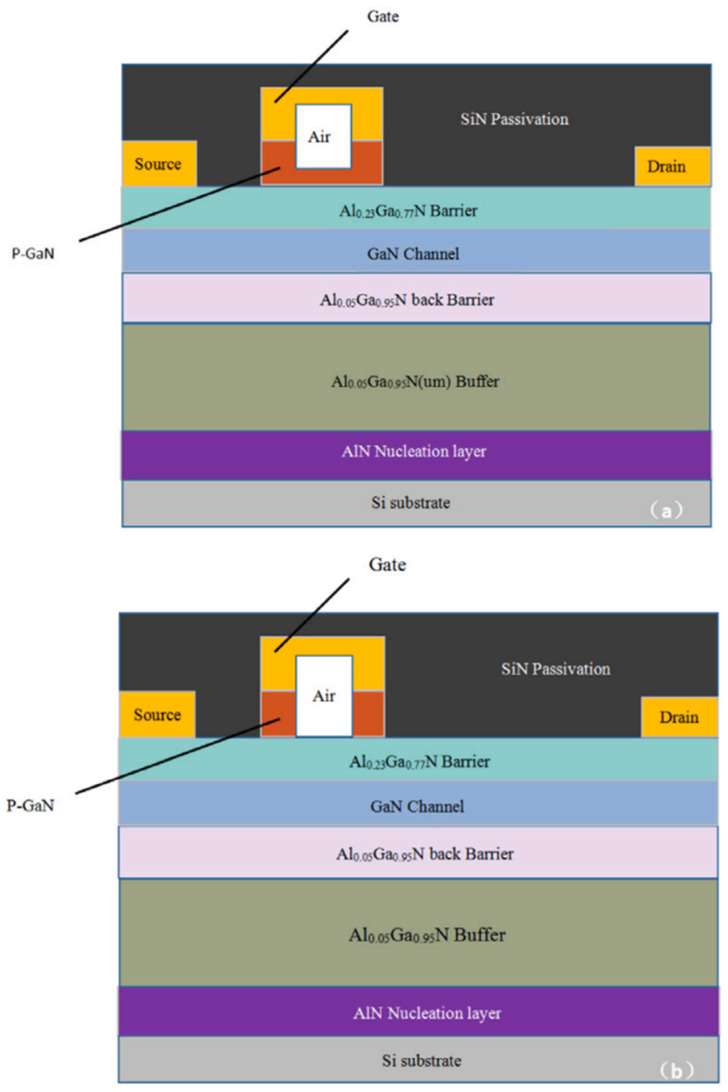
Schematic cross-sections of (**a**) AC-PC HEMT and (**b**) AC-PS HEMT [45].

In this paper, a review of previous research is presented. The *V*_TH_ shift in GaN HEMT devices with Schottky-type and ohmic-type gates at different temperatures is investigated. At elevated temperatures, ohmic-gated devices show a negative *V*_TH_ shift, which is attributed to the decrease in the inflection point voltage at the ohmic metal/p^+^-GaN interface. As the temperature increases, a positive *V*_TH_ offset occurs in devices with Schottky-type gates. This is due to the increase in gate current at high temperatures, which causes a rise in voltage across the Schottky metal/p-GaN junction. The time-resolved threshold voltage instability in Schottky-type p-GaN gate HEMTs has been investigated under HTFB and HTRB conditions. Under HTFB conditions, the negative shift in *V*_TH_ mainly comes from the tunneling effect induced by holes and is independent of temperature. Under HTRB conditions, the positive shift in *V*_TH_ is temperature-dependent due to electron trapping by defects created by hole emission and hot holes. The choice of gate metal material can be further optimized next, aiming to balance the relationship between output characteristics, *V*_TH_, and reliability, which can achieve a greater breakthrough in device performance [45]. In Figure 19a, a proposed air-cavity-P-GaN connected HEMT is shown. In Figure 19b, an air-cavity-P-GaN separated HEMT is shown.

## Figures and Tables

**Table 1:** Gate characteristics of different GaN devices.

Manufacturer	*V*_TH_ (V)	*V*_GSmin_~*V*_GSmax_ (V)	Gate Drive Voltage (V)
EPC [21]	1.4	−4~6	4~5
Panasonic [22]	1.2	−10~4.5	3~5
GaN System [23]	1.3	−10~7	5~6.5

## Data Availability

The data presented in this study are available upon request from the corresponding author.
